# Gene therapy in glaucoma-part I: Basic mechanisms and molecular genetics

**DOI:** 10.4103/0974-620X.60013

**Published:** 2010

**Authors:** Mohamed A. E. Soliman Mahdy

**Affiliations:** Department of Ophthalmology, Rustaq Hospital, Sultanate of Oman, Al-Hussein University Hospital, Faculty of Medicine, Al-Azhar University, Cairo - Egypt

**Keywords:** Glaucoma, gene therapy, genetic diagnosis of glaucoma

## Abstract

Glaucoma is the second most common cause of blindness in the world as determined by the World Health Organization (WHO). Glaucoma diagnosis, identification of people at risk, initiation of treatment and timing of surgical intervention remains a problem. Despite new and improving diagnostic and therapeutic options for glaucoma, blindness from glaucoma remains a major public health problem. The role of heredity in ocular disease is attracting greater attention as knowledge and recent advances of Human Genome Project and the HapMap Project have made genetic analysis of many human disorders possible.

Glaucoma offers a variety of potential targets for gene therapy. All risk factors for glaucoma and their underlying causes are potentially susceptible to modulation by gene transfer. The discovery of genes responsible for glaucoma has led to the development of new methods of Deoxyribonucleic acid (DNA)-based diagnosis and treatment. As genetic defects responsible for glaucoma are identified and the biochemical mechanisms underlying the disease are recognized, new methods of therapy can be developed. It is of utmost importance for ophthalmologists and glaucoma specialists to be familiar with and understand the basic molecular mechanisms, genes responsible for glaucoma and the ways of genetic treatment.

**Method of Literature Search:**

The literature was searched on the Medline database using the Pubmed interface.

## Article Outline

**PART 1**

**A] Introduction**

**B] Basic Molecular Genetics**

 DNA, Genes and Chromosomes

 Structure of DNA

 Gene Structure

 Genetic Code and Gene Expression

 Gene Expression

 Mutations

 Human Genome

 Gene Organization in Human Cells

 DNA-Based Diagnosis

**PART II**

**C] Genetic Etiology of Glaucoma**

**A. Childhood Glaucoma**

 *I. Congenital Glaucoma*

 *II. Anterior Segment Dysgenesis Syndromes*

  1. Axenfeld-Rieger

  2. Nail-patella Syndrome

  3. Aniridia and PAX6 Gene

**B. Primary Open -Angle glaucoma (POAG)**

 *I. Genetic Loci for POAG:*

  1. GLC1A (TIGR/Myocilin)

  2. GLC1B

  3. GLC1C

  4. GLC1D

  5. GLC1E

  6. GLC1F

  7. GLC1G

  8. GLC1J , GLC1K and other loci

II. Candidate Genes Causative of POAG

III. Genes Associated with POAG and Gene-Gene Interaction

**C. Pigment Dispersion Syndrome**

**D. Pseudoexfoliation Syndrome and Glaucoma**

**PART III**

**D] Conclusion: Developing a Diagnostic Panel for Patients at Risk for Glaucoma**

## Introduction

Glaucoma is a progressive optic neuropathy. It is characterized by morphologic changes of cupping of the optic nerve head, changes in the retinal nerve fiber layer and subsequently followed by characteristic visual field loss.[1,2] The final common pathologic event is retinal ganglion cell (RGC) death.

Primary open angle glaucoma (POAG) is a major public health problem. It is a slowly progressive eye condition that, once diagnosed requires lifelong observation and management. Even with careful control of intra-ocular pressure, 25-30% of patients continuously lose their visual fields.[3–8]

POAG is the most prevalent type of glaucoma in Western countries.[9] Around 2% of people aged more than 40 years (0.4% to 3.3%) are affected and the prevalence rises with age to 5% in those above 80 years.[10] Its frequency is four times higher in African Americans and African Caribbean.[11–14] In Oman, the rate of glaucoma cases reported by ophthalmologists was 1.14/1000; 11.5% of estimated blindness in Oman was due to glaucoma.[15,16]

Family history of primary open angle glaucoma (POAG) has long been recognized as a major risk factor implicating that hereditary factors and specific gene defects plays an important rule in its development[17,18] In one study, the prevalence of POAG was 7-10 times higher in first-degree relatives of patients than in the general population.[19] One-third of patients with POAG have IOPs within the normal range (normal-tension glaucoma (NTG).[20] Both high tension glaucoma (HTG) and NTG involves optic nerve loss. Genetic susceptibility is believed to underlie both types of glaucoma.

Despite new and improving diagnostic and therapeutic options for glaucoma, blindness from glaucoma is increasing and glaucoma remains the second most common cause of blindness in the world as determined by the WHO.[12–14,21,22]

The role of heredity in ocular disease is attracting greater attention as the knowledge and recent advances of Huan Genome Project and the HapMap Project made genetic analysis of many human disorders possible (the Human Genome was completed in April 2003 and the International HapMap Project in 2005).[23–25]

Glaucoma offers a variety of potential targets for gene therapy. Various causes of trabecular dysfunction and alterations in aqueous production reside in the anterior segment. Events leading to RGC damage and death are targets for genetic modulation. Several other hereditary disorders are also associated with glaucoma.[26–29] These risk factors and their underlying causes are potentially susceptible to modulation by gene transfer. As genetic studies continue, additional targets are likely to be identified.[30]

The discovery of genes responsible for glaucoma is opening the possibility of new methods of DNA-based diagnosis and treatment. As genetic defects and biochemical mechanisms responsible for glaucoma are, new methods of therapy can be developed. In some cases, understanding the responsible biochemical abnormalities may indicate metabolites, either small molecules or enzymes that could be administered to correct the problem. Using new technologies, novel methods of diagnosis and treatment, based on the genetic defects responsible for glaucoma, will allow individuals at risk for the disease to be identified and successfully treated before irreversible damage to the nerve has occurred.[31]

As large numbers of patients with genetic ocular disease, especially glaucoma, are identified, public demands for prevention, treatment and counseling increase. Consequently ophthalmologists, like other medical specialists are expected to be familiar with the clinical manifestations, transmission patterns, diagnostic techniques, and therapeutic modalities of heritable disorders in their specialty.[32]

## Basic Molecular Genetics

### Deoxyribonucleic acid, genes and chromosomes

The genetic information encoded as DNA in each cell directs development and regulates cellular growth, normal physiologic cellular functions necessary to sustain life and function in all human tissues. It is dependent on the activities of specific protein molecules. In turn, protein activity is dependent on the expression of the genes that contain the correct DNA sequence for protein synthesis.

Human DNA is packaged as chromosomes located in the nuclei of cells. Chromosomes are composed of individual strands of DNA wound about proteins called *Histones*. The entire collection of human chromosomes, called the *human genome*. It includes 22 paired autosomes and two sex chromosomes. Women have two copies of the X chromosome, and men have one X and one Y chromosome.[33,34]

### Structure of Deoxyribonucleic Acid

Chromosomes are composed primarily of proteins and DNA. The DNA molecule is a double-stranded helix. The backbone of each chain is formed by sugar-phosphate molecules. Each strand is composed of a sequence of a chain of Deoxyribose molecule linked by phosphate to each other and to which one of the nitrogen bases are attached. The nitrogen bases are either *Purine base*: Adenine (A) and guanine (G) or *Pyrimidine base*: Cytosine (C), and thymine (T). The sugar-phosphate unit together with the nitrogen base is called the *nucleotide*. Each nitrogen base can form a hydrogen bond with only one of the other bases to form a *base pair* (bp). The order of the bases in the DNA sequence forms the genetic code that directs the expression of genes.[33,35]

The double-stranded helix is formed as a result of hydrogen bonding between the nucleotide bases of opposite strands.[35] The bonding is specific, such that; A always pairs with T, and G always pairs with C. The specificity of the hydrogen bonding is the molecular basis of the accurate copying of the DNA sequence that is required during the processes of DNA replication (necessary for cell division) and transcription of DNA into RNA (necessary for gene expression and protein synthesis). It should be noted that the two DNA strands are complementary to each other so that each strand contains the information in a reverse sequence to the other strand. If the sequence of one strand is known, the sequence of the other could be predicted.[36]

### Gene Structure

A gene is part of the DNA that directs the sequencing of polypeptide chains (protein), ribosomal RNA (rRNA), or other gene products and its associated regulatory sequence. They are the determinants of heredity that are discrete entities that maintained their identity from one generation to the next. All genetic material has two critical functions, self duplication and information storage.[37]

Genes are the fundamental units used in the study of inherited disease, and occupy specific positions on chromosomes known as loci. The term gene can refer to the position on a chromosome (a locus) that governs a heritable trait, or to a form of the DNA sequence at the locus (an allele) that is associated with a particular phenotype.[37]

The genes are located in a linear order along the chromosome, each gene having a precise location or locus. Each of a pair of chromosomes (*Homologous chromosomes*) carries matching genetic information, i.e. they have the same sequence of gene loci. However, at any locus they may have slightly different forms which are called *alleles*.[38] The gene itself consists of both coding regions (exons) and variable-length intervening regions (introns). There are also enhancer regions which carry out regulatory activities particularly the expression of genes in tissues.[38] The genetic structure can be divided into the so -called 5′ untranslated regions (which contain the promoter) and regulatory regions (enhancer, inducer, and inhibitor regions). They serve as target for transcription factors, which activate genes and regulate and help determine whether the gene is transcribed. The promoter, which is under tissue specific control, turns the gene on (allows transcription and translation).[36]

### Genetic Code and Gene Expression

Genetic information is stored within the DNA molecule in the form of a *triplet code*, which is a sequence of three nucleotide bases which will translate through the mRNA to amino acid. Each triplet of nucleotide bases, which codes for a single amino acid is called a *codon*, it is the basic unit of genetic code.[36]

Gene expression begins with the recognition of a particular DNA sequence, called the promoter sequence, as a start site for RNA synthesis by the enzyme RNA polymerase. The RNA polymerase "reads" the DNA sequence and assembles a strand of RNA that is complementary to the DNA sequence (*transcription*). RNA is a single-stranded nucleic acid composed of the same nucleotide bases as DNA, except that Uracil takes the place of thymine. Every base in the mRNA molecule is complementary to a corresponding base in the DNA of the gene. The mRNA then migrates out of the nucleus into the cytoplasm where it becomes associated with the ribosomes, which are the site of protein synthesis where mRNA forms the template or mould for producing particular amino acids. Thus it is called template-RNA (tRNA). Genetic information from mRNA is translated into amino acids which are then incorporated in the polypeptide chain or protein synthesis. The sequence of bases in the mRNA directs the order of amino acids that make up the polypeptide chain. Transfer RNA (tRNA) molecules bind specific amino acids and recognize the corresponding three-base codon in the mRNA.

**Figure 1:**
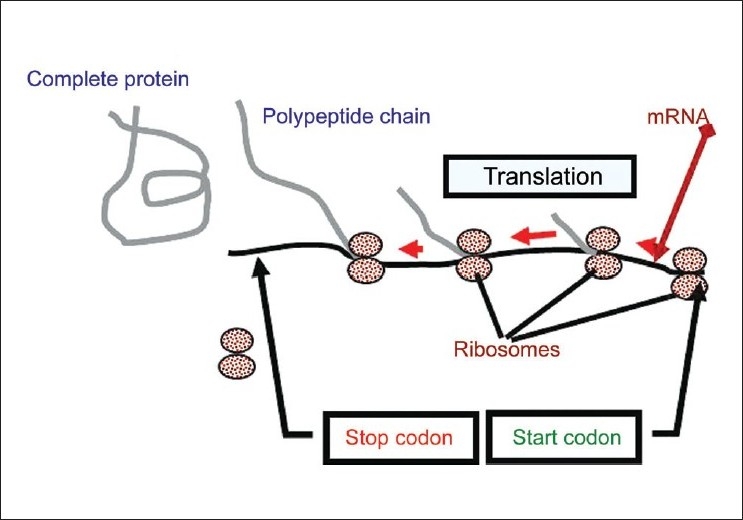
Diagrammatic representation of initiation and termination of the translation process of mRNA in the cytoplasm

In the cytoplasm, cellular organelles called ribosomes bind the mRNA in such a configuration that the RNA sequence is accessible to tRNA molecules and translate the code (codon) in the coding region of mRNA molecules into one specific amino acid which are aligned and joined to form the polypeptide. The codon ATG, which specifies the amino acid methionine, is the only codon used by the ribosome to initiate translation (*start or initiation codon*). Then, every three bases specify one amino acid attached without skipping or overlapping of codons until one of the codons TAG, TGA or TAA is encountered. These three codons are called *stop or termination codons* because any one of them serves terminate the translation [Figure 1].[37] Since a polypeptide chain is determined by the sequence of triplets that makes up a segment of DNA, and some polypeptide serves as structural components of the organism, and others function as enzymes, so it is obvious how the triplet code of DNA is responsible for the total metabolic activity of the cell and its parent organism.[34,39,40]

### Mutations

These are changes in the gene DNA sequence, due to alteration in the sequence of bases - deletion or insertion of DNA materials at a particular point. So, a different amino acid is substituted in the protein molecule giving either abnormal protein or enzyme. This results in a biologically significant change in the function of the encoded protein. These changes result in two possibilities; (1) no active gene product being produced (*null mutation*), (2) gain of function which might be detrimental (leading to disease) or beneficial (leading to evolution).[36]

If a particular gene is mutated, the protein product might not be made, or it might be produced but work poorly. In some cases, mutations create proteins that have an adverse effect on the cell (*dominant negative effect*). Point mutations (the substitution of a single base pair) are the most common mutations encountered in human genetics. *Missense mutations* are point mutations that cause a change in the amino acid sequence of the polypeptide chain. The severity of the missense mutation is dependent on the chemical properties of the switched amino acids and on the importance of a particular amino acid in the function of the mature protein. Point mutations may also decrease the level of polypeptide production because they interrupt the promoter sequence, splice site sequences, or create a premature stop codon.[40]

### The Human Genome

It is a set consisting of one of each autosome as well as both sex chromosomes; it includes one copy of every human locus. The chromosomal molecules of DNA from one human genome contain a sequence of approximately 3.2 billion base pairs, with about 60.000 to 100.000 genes, embedded in this DNA sequence in average for one gene about 30.000 base pairs.[37] The Human Genome Project that had been finished in 2003 aimed at identifying all the genes in human DNA and determining the sequences of the three billion base pairs that make up human DNA.[41] The HapMap Project′s goal was to develop a haplotype map of the human genome, and describes the common patterns of human DNA sequence variation, thus providing a necessary infrastructure for single nucleotide polymorphism-based genetic analyses.[42,43]

### Gene organization in human cells

**Figure 2:**
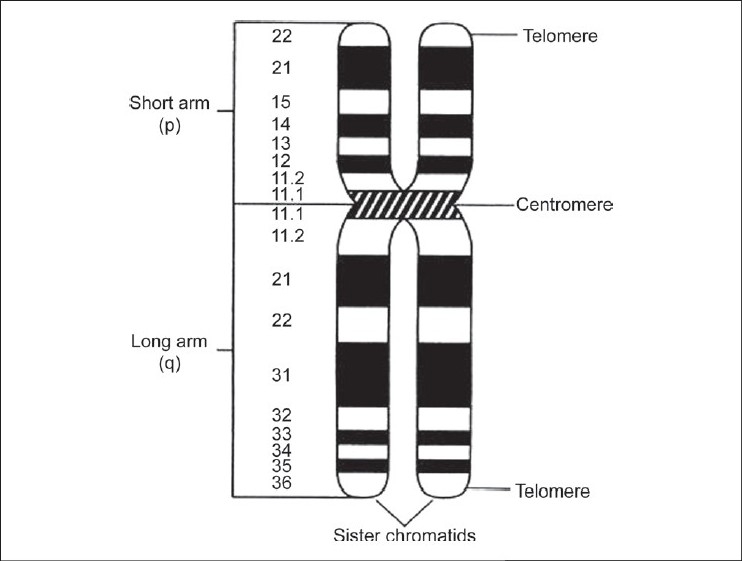
Anatomy and banding of human chromosome

Each chromosome has a centromere that divides it into two arms, the short arm is called "*P*" arm and the long arm is called "q" arm. A chromosome with a very small short arm is called acrocentric chromosome [Figure 2].

There is now a standardized nomenclature for the set of darkly and lightly staining bands characteristic of each human chromosome arm.[37–44] An example is a systemic abnormalities involving chromosome 11p13 deletion, causing aniridia and elevated predisposition to Wilm′s tumour. 11 refer to the number of the chromosome, *P* refers to the short arm, and 13 refer to the chromosomal segment or band carrying the gene.

### Deoxyribonucleic acid-Based Diagnosis

The use of molecular tools to demonstrate causative DNA mutations and identify individuals at risk for an inherited condition is called DNA-based diagnosis.[45] The goal of genetic diagnosis is early recognition of a disease so that intervention can be undertaken to prevent or reverse the disease process. This was one of the goals of the Human Genome Project. Two general approaches have been used to detect mutations in genes. The indirect approach uses genetic linkage analysis, and the direct approach identifies specific changes in DNA sequence.[40]

### Linkage analysis

Can be used to diagnose any genetically mapped disorder. Segregation of genetic markers known to be linked to a gene responsible for a condition is used to determine whether an individual has inherited a chromosome that carries the abnormal gene. This method does not require physical isolation and sequencing of the gene.

### Direct mutation analysis

Uses a variety of techniques based on the *DNA sequence* of a gene to identify the specific base-pair change that is responsible for the disease. The gene responsible for the disease must first be isolated and sequenced.[40] In some disorders, the majority of stricken individuals are affected by the same mutation. For disorders of this type, a simple screening test based on the particular mutation may be developed.
